# Genome Report: Identification and Validation of Antigenic Proteins from *Pajaroellobacter abortibovis* Using *De Novo* Genome Sequence Assembly and Reverse Vaccinology

**DOI:** 10.1534/g3.116.036673

**Published:** 2016-12-28

**Authors:** Bryan T. Welly, Michael R. Miller, Jeffrey L. Stott, Myra T. Blanchard, Alma D. Islas-Trejo, Sean M. O’Rourke, Amy E. Young, Juan F. Medrano, Alison L. Van Eenennaam

**Affiliations:** * Department of Animal Science, University of California, Davis, California 95616; † Department of Pathology, Microbiology and Immunology, School of Veterinary Medicine, University of California, Davis, California 95616

**Keywords:** epizootic bovine abortion, *Pajaroellobacter abortibovis*, reverse vaccinology, recombinant vaccine, *Ornithodoros coriaceus*

## Abstract

Epizootic bovine abortion (EBA), or “foothill abortion,” is the leading cause of beef cattle abortion in California and has also been reported in Nevada and Oregon. In the 1970s, the soft-shelled tick *Ornithodoros coriaceus*, or “pajaroello tick,” was confirmed as the disease-transmitting vector. In 2005, a novel Deltaproteobacterium was discovered as the etiologic agent of EBA (aoEBA), recently named *Pajaroellobacter abortibovis*. This organism cannot be grown in culture using traditional microbiological techniques; it can only be grown in experimentally-infected severe combined immunodeficient (SCID) mice. The objectives of this study were to perform a *de novo* genome assembly for *P. abortibovis* and identify and validate potential antigenic proteins as candidates for future recombinant vaccine development. DNA and RNA were extracted from spleen tissue collected from experimentally-infected SCID mice following exposure to *P. abortibovis*. This combination of mouse and bacterial DNA was sequenced and aligned to the mouse genome. Mouse sequences were subtracted from the sequence pool and the remaining sequences were *de novo* assembled at 50x coverage into a 1.82 Mbp complete closed circular Deltaproteobacterial genome containing 2250 putative protein-coding sequences. Phylogenetic analysis of *P. abortibovis* predicts that this bacterium is most closely related to the organisms of the order Myxococcales, referred to as Myxobacteria. *In silico* prediction of vaccine candidates was performed using a reverse vaccinology approach resulting in the identification and ranking of the top 10 candidate proteins that are likely to be antigenic. Immunologic testing of these candidate proteins confirmed antigenicity of seven of the nine expressed protein candidates using serum from *P. abortibovis* immunized mice.

EBA, or “foothill abortion,” has affected the California beef cattle industry for over 60 yr. It is a specific fetal syndrome with distinctive pathology characterized by late-term abortion or birth of weak calves (Howarth *et al.* 1956). In the 1970s, the soft-shelled tick *Ornithodoros coriaceus*, or “pajaroello tick,” was shown to have the same geographic distribution as EBA and was confirmed to be the EBA-transmitting vector (Schmidtmann *et al.* 1976). EBA cases have historically been confined to California, Nevada, and Oregon (Teglas *et al.* 2006).

Diagnosis is based upon the presence of characteristic gross and microscopic lesions of the aborted fetus, increased fetal serum immunoglobulin concentrations, history of exposure to the tick vector, and elimination of other causes (Kennedy *et al.* 1983). The inability to experimentally propagate the etiologic agent resulted in many failed attempts to identify the causative microbe. Suppression hybridization polymerase chain reaction (PCR) ultimately identified the causative agent of EBA (aoEBA) as a novel Deltaproteobacterium closely related to organisms in the order Myxococcales (King *et al.* 2005). Analysis indicated that the most closely related fully-sequenced organism is *Sorangium cellulosum*; however, updated 16S rRNA sequence analysis groups it into a novel clade with unnamed, uncultured environmental bacteria (Brooks *et al.* 2016).

Identification of this Deltaproteobacterium, *Pajaroellobacter abortibovis*, allowed for the development of new diagnostic methodologies (Blanchard *et al.* 2010, 2014; Brooks *et al.* 2011). The visualization of *P. abortibovis* through immunohistochemistry and DNA-based diagnostic techniques using PCR have increased diagnostic sensitivity (Anderson *et al.* 2006; Brooks *et al.* 2016). The inability to culture this bacterium in media necessitated an alternate host for further EBA research. SCID mice have become the preferred host for propagation of *P. abortibovis*, providing a consistent bacterial load for experimental analysis (Blanchard *et al.* 2010).

A live bacterial vaccine consisting of *P. abortibovis*-infected SCID mouse spleen cells is undergoing field trials with favorable results (J. L. Stott, unpublished data). However, the vaccine can only be administered to nonpregnant cows as the bacteria in the vaccine are live and virulent. A recombinant vaccine would provide the added benefit of being safe for administration to both open and pregnant cattle (Ellis 1999). Since recombinant vaccine development relies on a thorough understanding of the pathogen’s genomic makeup, sequencing and assembly of the *P. abortibovis* genome was the first step in the development of a recombinant vaccine for EBA.

Next-generation sequencing (NGS) has increased access to large quantities of sequence data, thereby enabling the successful assembly of many bacterial genomes. Since the advent of NGS, a new field known as “Reverse Vaccinology” (RV), defined as an *in silico* approach to discover potential vaccine candidates starting with the genome sequence of a bacterial strain, has emerged (Rappuoli 2001). RV predicts putative antigens based on genomic sequence data and circumvents the limitations of traditional vaccine development for pathogens that are difficult to cultivate. The identification of vaccine candidates through an *in silico* approach can substantially reduce the immunologic bench experiments required to identify potential vaccine candidates.

The objectives of this study were to assemble and annotate the complete genome of *P. abortibovis*, use a RV approach to identify vaccine candidates, and perform immunologic western blots to experimentally evaluate the antigenicity of these candidate proteins.

## Materials and Methods

### Organism derivation

Spleen tissue was collected from two EBA-infected BALB/c *scid* mice for DNA extraction (Blanchard *et al.* 2010). Spleens were first pressed through 100 µm Falcon nylon mesh cell strainers (Corning Inc., Corning, NY) with Dulbecco’s phosphate buffered saline (DPBS). The preparations were pelleted by centrifugation at 300 × *g*, the supernatant harvested, and centrifuged at 1550 × *g*. The resulting bacterial pellet was suspended in DPBS with 10% DMSO, rate frozen, and stored in liquid nitrogen. DNA was extracted using standard phenol:chloroform methods (Sambrook and Russell 2006). Spleens from two EBA-infected BALB/c *rag* mice where minced and stored in RNA*later* (ThermoFisher Scientific, Rockford, IL). RNA was extracted using the ZR-Duet DNA/RNA mini-prep kit (Zymo Research Corporation, Irvine, CA).

### Ethics statement

Mice were housed at the University of California, Davis (UCD) and studies were conducted in compliance with the U. S. Federal Animal Welfare Act and the Health Research Extension Act, and overseen by the UCD Institutional Animal Care and Use Committee (Protocol #17358, Approved 12/13/2012).

### Sequencing

The genomic library was prepared using the NEBNext DNA Library Prep Kit (New England BioLabs, Ipswich, MA) with an average fragment size of 230 bp. The library was sequenced using 100 bp paired-end reads (PE100) on the Illumina HiSeq 2500 (Illumina, San Diego, CA). A mate-pair genomic library was prepared using the Nextera Mate-Pair Protocol (Illumina) with an average fragment size of 2500 bp, and was paired-end sequenced with 100 bp reads on the Illumina HiSeq analyzer. Ribosomal RNA was removed from total RNA using the RiboMinus Invitrogen Eukaryote kit for RNA (Life Technologies, Carlsbad, CA). The RNA-Seq library was prepared using the NEBNext Ultra RNA Library prep kit (New England BioLabs) with an average fragment size of 250 bp, and was paired-end sequenced with 100 bp reads on the Illumina HiSeq analyzer.

### Data processing

**Figure 1:**
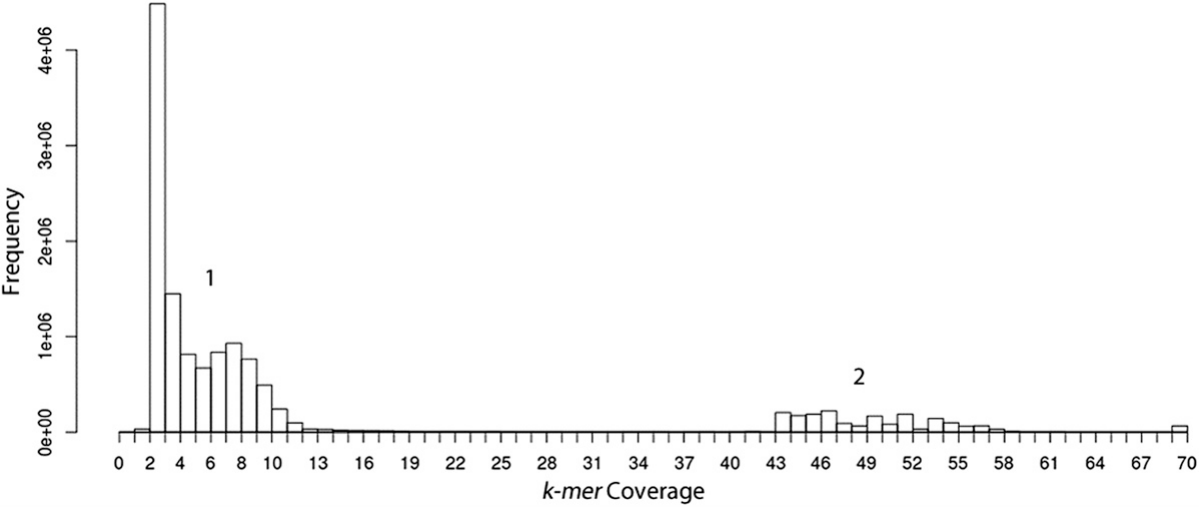
Frequency of coverage for Velvet assembly with hash length 31. The first peak in the bimodal distribution (“1”) represents the murine contigs that had lower coverage due to the larger genome size. The smaller peak (“2”) represents the frequency of assembled contigs originating from *P. abortibovis*. These contigs were collected for further assembly with the average coverage parameter set to 50.

Genomic sequences from the paired-end library were aligned with high stringency to the murine genome (mm10) (Mouse Genome Sequencing Consortium 2002) with Bowtie2 (Langmead and Salzberg 2012) and unaligned reads were collected. Remaining reads were assembled utilizing a de Bruijn graph genome assembler, Velvet (Zerbino and Birney 2008), with a k-mer length of 31. The k-mer coverage for the resulting contigs were plotted on a histogram to determine the frequency of coverage (Figure 1). The assembly was optimized utilizing only k-mer coverages consistent with the bacterial genome sequence, thereby further reducing the murine read contamination. The final Velvet assembly parameters consisted of an expected coverage of 50, minimum coverage of 25, maximum coverage of 70, and an insert length of 230. Eleven contiguous sequences of assembled reads (contigs) with similar depth of coverage were collected from Velvet, and nucleotide comparisons were made to the NCBI genome database to ensure that contigs were not derived from the mouse genome.

Mate-pair library reads were aligned to the Velvet contigs using Bowtie2, and the resulting sequence alignment map (SAM) output was used for manual contig scaffolding. To determine scaffold size and validate contig orientation, the stand-alone scaffolder SSpace (Boetzer *et al.* 2011) was used. The scaffolded gaps from the assembled contigs were decreased by the stand-alone gap filling program GapFiller (Nadalin *et al.* 2012). Correct contig orientation and position within the circular genome was determined by mate-pair mapping events, where one mate mapped to one contig and the other mate mapped to a different contig. The position and orientation of the contigs was confirmed and unassigned base pairs were utilized to fill the sequence gaps.

All contiguous sequences were compared using Blast2Seq (Tatusova and Madden 1999) to visualize the consistency of assemblies at each stage using different assembly parameters.

### Sanger sequencing

Unassigned base pairs remaining at the end of the *de novo* assembly were visualized using Geneious (Kearse *et al.* 2012) and segments of DNA were identified that contained regions of assembled sequence with multiple unassigned base pairs (N’s). PCR primers (Table 1) were designed to span these blocks of unassigned base pairs for an optimal amplicon size of 450 bp using the program Primer3 (Untergasser *et al.* 2012). An additional nested primer was designed for one fragment that had a larger amplicon size, and the nested PCR product was utilized for Sanger sequencing.

**Table 1:** Sequences of primer pairs for Sanger sequencing

Gap	Primer Sequence (5′–3′)	Amplicon Size (bp)
1	G1F TCGGTCGAATACTCCGAAAG	447
	G1R CATGGGACTGTTCTGGACCT	
2	G2F CACTGCCAAATGTCAAAGCA	436
	G2R GGAGCGAGTATTGGCATTGT	
3	G3F GCGTATCCGTCAAGATTGGT	844; 601*^a^*
	G3R1 TACTCGGTGGCCTTGCTATT	
	G3R2 CTCAGGCTCGTTGGCTTATG	

a Amplicon size from primer G3F to G3R1 is 844 bp, and the amplicon size from G3F to G3R2 is 601 bp.

For PCR analysis, two DNA templates that had been isolated from *P. abortibovis* were utilized (60 and 400 ng). One microliter of each DNA template was amplified using GoTaq Green Master Mix (Promega, Madison, WI). Cycling conditions consisted of an initial 95° denaturation for 2 min, followed by 35 cycles of 95° for 30 sec, 58° for 30 sec, and 72° for 30 sec, and a final extension of 72° for 5 min. PCR products (15 µl) were purified using the QIAquick PCR purification kit (QIAGEN, Valencia, CA).

Sanger sequencing was performed by Davis Sequencing on an Applied Biosystems 3730 DNA Analyzer (Life Technologies). Chromatogram visualization and trimming of DNA sequence were performed with ChromasPro software (Technelysium, Queensland, Australia) and curation of a consensus sequence through visualization was performed using Geneious software (Kearse *et al.* 2012).

The chromatograms displayed a pattern in which overlapping peaks were present following a stretch of homopolymeric base pairs. The template with the largest signal for each region was selected as the consensus sequence and was compared to the NGS sequence reads. This is indicative of “enzyme slippage” that commonly occurs during clonal amplification, such as with Illumina HiSeq NGS sequencing. Visualization of the NGS sequencing assembly supported this hypothesis because the coverage level dropped significantly and sequencing reads were shorter at the homopolymeric sections, likely as a result of the polymerase not being able to extend through these regions. Although it was not possible to confirm the exact number of nucleotides in the homopolymers, evidence-based predictions were used to close the homopolymeric gaps in the assembly.

### Annotation

The assembled genome for *P. abortibovis* was annotated using the Rapid Annotation using Subsystem Technology (RAST) (Aziz *et al.* 2008). RAST is a fully automated annotation engine designed for annotation of bacterial genomes. Any gene candidate without a function was further searched for similarity with the top 30 closest phylogenetic relatives using BLASTP. Gene candidates that were <90 bp in length were removed from further analyses.

### Comparative genome analysis

Comparative sequence analysis was performed between *P. abortibovis* and *S. cellulosum* using the “sequence-based comparison tool” in the SEED viewer (Overbeek *et al.* 2014), which is a BLAST-based tool to compare two genomes. The functional comparison was performed using the “function-based comparison tool” in SEED viewer, which assesses the presence and absence of functional roles that have been linked to subsystems in RAST. The number of genes contributing to each functional category was quantified and the percent loss of genes contributing to that functional category was computed from *S. cellulosum* to *P. abortibovis*.

Comparative analyses of specific genes present in *P. abortibovis* from *Myxococcus xanthus*, *S. cellulosum*, and *Lawsonia intracellularis* were performed using BLAST2seq. To perform the analysis, the genes were extracted from the PATRIC database and nucleotide-based alignment was performed against the assembled *P. abortibovis* genome. Subsequent amino acid (AA) alignment was performed to identify the presence of proteins with AA sequence variants.

### Reverse vaccinology

#### Gene prediction:

Initial protein coding gene prediction was performed by the RAST annotation pipeline, which utilizes Glimmer3 (Delcher *et al.* 2007). Gene prediction was also performed using Prodigal (Hyatt *et al.* 2010), GeneMarkS (Besemer *et al.* 2001), and the NCBI prokaryotic genome annotation pipeline (Tatusova *et al.* 2013, 2016), to test the effect of gene prediction on candidate selection. Thereafter, the reverse vaccinology protocol described below was carried out with proteins predicted by all four gene prediction programs.

#### Vaxign:

A comprehensive reverse vaccinology program, Vaxign (He *et al.* 2010), was utilized for analysis alongside supplementary stand-alone programs. The pipeline includes protein subcellular localization (SCL), transmembrane (TM) helices, adhesin probability, and protein conservation to human, pig, and mouse proteins. Further description of individual programs utilized in Vaxign are in subsequent sections.

#### SCL:

SCL of gram-negative bacteria predicts whether proteins are localized to the cytoplasm, inner membrane, periplasm, outer membrane, or are extracellular (*i.e.*, secreted). The predictions were determined using PSORTb2.0 in Vaxign and three additional stand-alone software programs: PSORTb3.0 (Yu *et al.* 2010), SOSUI-GramN (Imai *et al.* 2008), and CELLO (Yu *et al.* 2006). No difference was observed between PSORTb2.0 and PSORTb3.0, so results from PSORTb2.0 were excluded from further analysis. A score of 1 was assigned for prediction of outer membrane or extracellular, 0.5 for periplasm, and 0 for cytoplasm and inner membrane. When more than one SCL was predicted, they were scored by summing individual scores (*S*) and dividing by the number of SCLs predicted (*n*) *$\sum S_{n}/n.$* The score for each of the three SCL programs was then summed for final a SCL score for each protein.

#### TM helix:

The Vaxign suite of tools utilizes the PROFtmb (Bigelow *et al.* 2004), a profile-based hidden Markov model-based software program to predict the number of TM helices in a bacterial protein. A maximum threshold of one TM domain is permitted for vaccine candidates.

#### Protein conservation:

OrthoMCL (Li *et al.* 2003) is used by Vaxign to compare the sequence against eukaryotic organisms for protein conservation between each protein and human, mouse, and pig proteins. The default *E*-value was set to 10^−5^ and was also utilized for BLASTP (Altschul *et al.* 1990) analysis against the bovine and *S. cellulosum* genomes. Conservation in eukaryotes and nonpathogenic prokaryotes was used to exclude any conserved protein from being a candidate for a recombinant vaccine. Two of the P. *abortibovis* candidates were removed for being highly conserved in eukaryotes. Four candidates were filtered based on homology with *S. cellulosum*.

#### Adhesin prediction:

Vaxign utilizes the program SPAAN (Sachdeva *et al.* 2005) for adhesin probability prediction and has a default value of 0.5 for the threshold. SPAAN utilizes the five protein attributes of adhesins, which include the AA frequencies, multiplet frequencies, dipeptide frequencies, charge composition, and hydrophobic composition, to determine the probability that a protein is an adhesin.

An adhesin is a bacterial cell surface component that facilitates adhesion to other cells and is essential for bacterial pathogenesis. Many adhesins are being evaluated for their ability to induce protective immunity. The program SPAAN that is carried out by Vaxign has been validated to be 97.4% accurate, 89% sensitive, and 100% specific at threshold 0.5 (Sachdeva *et al.* 2005). Therefore, this criterion was used to filter candidates. Only 2.7% (59 of 2174) of the total genes met the threshold and hence this step reduced the *P. abortibovis* candidates by almost two thirds, from 29 candidates to 10. After subcellular localization, the adhesin probability was the selection criterion that eliminated the most candidates.

#### Protective antigenicity:

VaxiJen (Doytchinova and Flower 2007) is a software program that predicts protective antigenicity through an alignment free approach based on AA properties. The default cutoff for protective antigenicity prediction is a probability of 0.4. However, a cutoff of 0.5 is optimal for bacterial prediction.

#### RNA sequencing analysis:

RNA sequencing reads were mapped to the assembled *P. abortibovis* genome using stringent parameters in Bowtie2 (Langmead and Salzberg 2012). These parameters allowed one mismatch in the 100 bp read. SAM files were used for input to the visualization program GenomeBrowse (www.goldenhelix.com, 2.0.0) and FPKM (fragments per kilobase of transcript per million mapped reads) values were obtained using Cufflinks (Trapnell *et al.* 2013).

#### Selection criteria:

The selection criteria utilized for vaccine candidate prediction were: (a) a subcellular localization score of ≥2, (b) ≤1 TM helix, (c) not conserved in any eukaryotes (human, mouse, pig, or cow), (d) not conserved in *S. cellulosum*, (e) adhesin probability >0.5, and (f) protective antigenicity prediction >0.5. RNA sequence expression with an FPKM > 0 was used for candidate ranking but absence of expression was not used as a criterion for elimination of candidates. The most highly transcribed gene, *BCY86_01995*, was expressed at a level that was an order of magnitude higher than the next most highly transcribed gene, *BCY86_08695*.

### Immunogenicity testing

Protein candidates were expressed by GenScript (Piscataway, NJ) utilizing *Escherichia coli* as an expression host. Codon optimization was performed for optimized expression of protein candidates. Proteins were purified using a 6x His tag. Protein preparations were mixed with sample application buffer lacking 2-mercaptoethanol (2-ME), loaded onto precast 4–20% Mini-Protean TGX gels (BioRad, Hercules, CA) at concentrations ranging from 0.1 to 1.0 μg/well and transferred to PVDF membranes with the Trans-Blot Turbo Mini PVDF Transfer Packs (BioRad). Membranes were blocked with SuperBlock T20 blocking buffer (ThermoFisher Scientific) and all washes were conducted with PBS containing 0.1% Tween 20. Blotted proteins were screened against one of the following: (a) mouse anti-6x His (GenScript), (b) pooled serum from BALB/c mice inoculated with *P. abortibovis*, (c) serum from a cow following a confirmed EBA abortion, (d) an aborted EBA-positive fetus, (e) commercially available mouse sera (Gemini Biosciences, West Sacramento, CA), (f) serum from a cow with no known *P. abortibovis* exposure, or (g) commercially available fetal bovine serum (Gemini Biosciences). Sera b, c, and d are referred to as immune sera and e, f, and g as EBA-negative controls. The presence of *P. abortibovis* antibody was confirmed in serum from *P. abortibovis*-exposed animals and absence in those with no known exposure by indirect fluorescent antibody test (IFAT) as previously described (Blanchard *et al.* 2014). Antibody binding was detected with alkaline phosphatase-conjugated anti-mouse IgG (H&L) (Vector Laboratories, Burlingame, CA) or anti-bovine IgG (H&L) (Jackson ImmunoResearch Laboratories, Inc., West Grove, PA) as appropriate, followed by NBT/BCIP substrate (Roche Diagnostics, Indianapolis, IN). The degree of antibody binding was subjectively assigned by visual appraisal of the developed membrane with a three-level scale. The scale ranged from no detected band (−), to faint bands (±), to distinct bands (+). Binding of anti-6x His identified the bands of interest, and color intensity served as the baseline for evaluating antibody binding when probed with murine or bovine serum samples.

### Data availability

Data supporting the conclusions of this article are available in GenBank, accession number CP016908. Gene names are those predicted by the NCBI Prokaryotic Genome Annotation Pipeline (Supplemental Material, Table S1). Table S1 summarizes the RNA-Seq results from the genes predicted by the NCBI Prokaryotic Genome Annotation Pipeline. Raw RNA-Seq data can be accessed through the NCBI Sequence Read Archive (SRA), accession number SRP095186. Figure S1 shows the western blot analyses for the top nine protein candidates.

## Results

### Genome assembly

Sequencing of the genomic library yielded a total of 243,271,075 100 bp paired-end (PE100) reads consisting of a combination of both mouse and bacterial DNA. To increase the concentration of bacterial reads within our pool of total reads, all reads were aligned to the mouse genome (mm10) (Mouse Genome Sequencing Consortium 2002) and the unmapped putative bacterial reads were collected for further analysis. Despite efforts to enrich for bacterial DNA, which included a variety of DNA extraction methods and cell filtering, 98.4% of the reads from the single-cell suspension mapped to the murine genome leaving only 4,030,414 (1.6%) nonmurine unmapped paired-end reads.

Sequences that did not map to the mouse genome were used as input for the Velvet de Bruijn graph assembler. The optimal assembly, defined as the largest N_50_, for the dataset was a k-mer length of 31 and k-mer coverage equal to ∼50 (Figure 1). The optimized Velvet assembly yielded 11 contigs with an average N_50_ of 400,733 bp, max contig length of 683,082 bp, and total assembly of 1,815,859 bp (Table 2). The resulting contigs were compared to the NCBI nucleotide database using nblast, and 10 of 11 contigs were found to be most similar to organisms of the class Deltaproteobacteria. One contig was most similar to the mouse genome, suggesting that mouse sequences remained in the data, and this contig was removed from further analysis.

**Table 2:** Assembly statistics for contiguous sequences at each stage of genome assembly

	Assembly Statistics			
Method	Contigs	Total bp	*N*_50_	Unassigned bp (*N*)
Velvet	11	1,815,859	400,733	1,661
SSpace	1	1,821,593	1,821,593	1,741
GapFiller	1	1,821,632	1,821,632	56
Sanger Seq	1	1,821,581	1,821,581	6

Contigs, number of contiguous sequences; Total bp, total number of base pairs assembled; *N*_50_, contig length when the sum of contigs is >50% of total assembly; Unassigned bp (*N*), number of unassigned base pairs; Seq, sequencing.

The 10 contigs were manually scaffolded by aligning all 23 million PE100 mate-pair reads (2675 bp insert size) to the assembled Velvet contigs using Bowtie2 and parsing the SAM file to pull out the 1725 mate-pair reads that aligned to two separate contigs. Once it was determined that all of the contigs were present to circularize the genome, the stand-alone scaffolder SSpace was used to confirm the manually curated scaffolds. The 63,642 (0.27%) mate-pair reads that aligned to the Velvet contigs were collected and utilized alongside the original paired-end reads that did not map to the murine genome for SSpace. The scaffolded assembly resulted in one contiguous 1,821,593 bp circular genome starting at the origin of replication (TTATCCACA). The scaffolded genome contained 1741 unknown bp, which equates to 0.096% of the genome or approximately one unknown nucleotide in every 1050 bp. Therefore, the assembled genome meets the standards for NCBI genome completion, which is one unknown nucleotide in each 1000 bp. A supplementary gap filling program, GapFiller, was used to decrease the number of unassigned base pairs and it was successful in producing a 1,821,632 bp genome with 56 unknown bp (Table 1).

The resulting genome contained three gaps consisting of multiple continuous unknown base pairs (24, 16, and 8 bp). These gaps were amplified using PCR and sequenced by Sanger sequencing. Each of the gaps was the result of a long homopolymeric stretch of DNA sequence which interfered with the Velvet assembly. Through the use of Sanger sequencing and visualization, the genome was further improved to a final size of 1,821,581 bp with 6 unknown bp (Table 1). This sequence has been deposited in GenBank (Accession Number: CP016908).

### Genome annotation

The Rapid Annotation using Subsystem Technology (RAST) pipeline resulted in the prediction of 2250 coding sequences that were assigned to 229 subsystems (a set of gene functions that are grouped together to implement a specific biological process). There were 47 noncoding RNAs predicted, with 44 of them being tRNAs and one copy each of 5S, 16S, and 23S ribosomal RNAs. The 2250 genes covered 1,544,122 bp of the 1,821,632 bp assembled, which equates to 84.8% of the genome covered by coding genes. The average length of genes predicted was 687 bp and ∼48.5% of the genes were identified from RNA-Seq data of spleen tissue from *P. abortibovis*-infected SCID mice. The initial prediction was only able to assign a putative function to 942 (42%) of the predicted genes and subsequent BLAST searches were able to assign putative functions to an additional 34 genes for a total of 976 (43%) genes with an assigned function. Hypothetical predicted proteins were generally shorter in length with an average gene length of 378 bp, and of these only 25% were represented in the RNA-Seq data. Of the 954 hypothetical genes that were not expressed, the average gene length was 218 bp (Table 3).

**Table 3:** Number of predicted proteins, gene length, and percent expression in predictions from the RAST gene prediction program

RAST Annotation	# of Predicted Genes	Average Gene Length (bp)	% Expressed (FPKM > 0)
Initial	2250	687	48.5
Hypothetical proteins	1274	378	25
Nonexpressed Hypo thetical proteins	954	218	—

RAST, Rapid Annotation using Subsystem Technology; FPKM, fragments per kilobase of transcript per million mapped reads.

**Figure 2:**
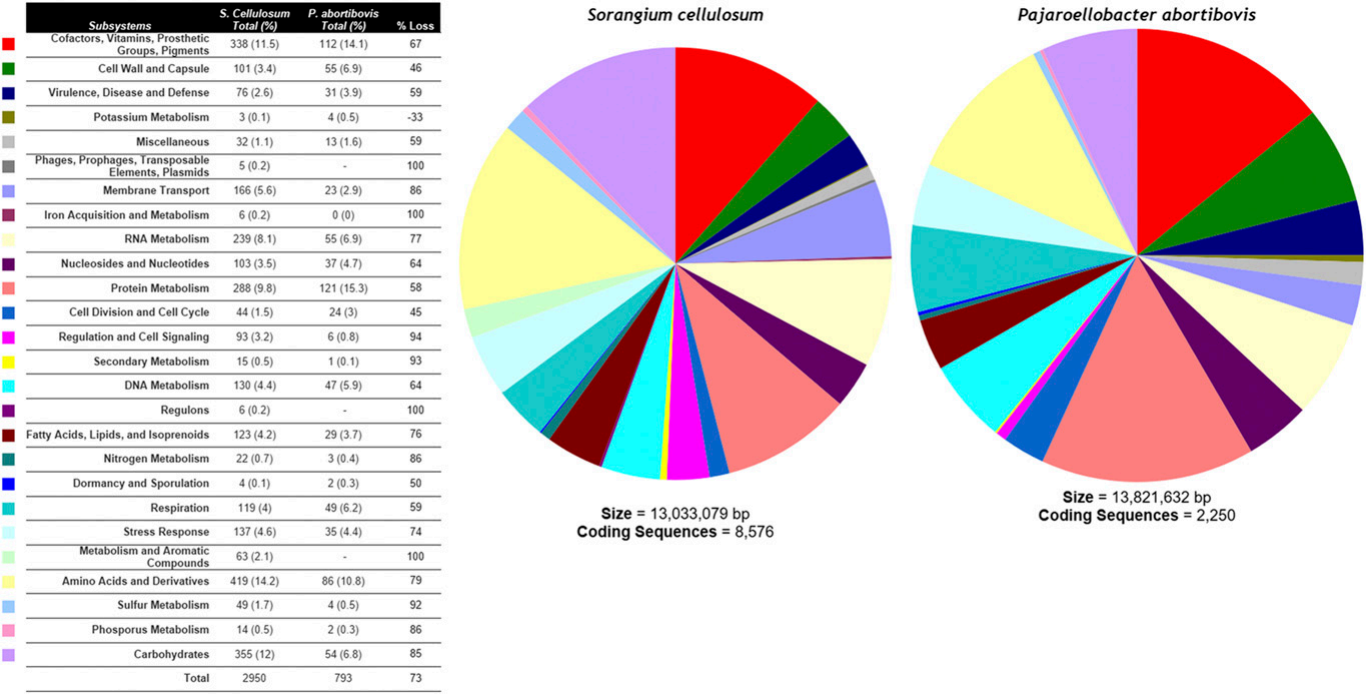
Analysis of functional group reduction between *S. cellulosum* and *P. abortibovis*.

The protein sequence-based comparison of *P. abortibovis* and *S. cellulosum*, the closest nonpathogenic phylogenetic relative to *P. abortibovis*, showed that 1143 (51%) of the predicted proteins shared >20% AA sequence homology, 465 (21%) of the predicted proteins shared >50% AA homology, and 14 (0.6%) of the predicted proteins shared >80% homology. RAST designates each gene into a hierarchy of categories starting with a specific gene function, which is in a subsystem, that is then in a subcategory and a category. As a result, multiple open reading frames (ORFs) may have the same functional annotation. Of the total 666 *P. abortibovis* gene functions, 620 (93%) were shared between the two organisms and only 46 functions were identified in *P. abortibovis* but not in *S. cellulosum*. The myxobacterium *S. cellulosum* had 667 unique functions that were not present in *P. abortibovis* (Figure 2).

Figure 2 displays the total number of ORFs for each subsystem grouped by functional category. This enables the calculation of the percentage of total genes lost in the evolution of *S. cellulosum* to *P. abortibovis* for each functional category. Overall, there was a 73% loss of genes in *P. abortibovis*. The percentage of genes lost (and gained) from each functional category varied widely, ranging from gaining one gene in potassium metabolism to losing all of the genes (100% loss) associated with three functional categories [(1) Phages, Prophages, Transposable elements, and Plasmids; (2) Regulons; and (3) Metabolism of Aromatic Compounds]. Annotations were submitted to the NCBI database using the NCBI Prokaryotic Genome Annotation Pipeline.

### Reverse vaccinology

All genes predicted for *P. abortibovis* were analyzed using a reverse vaccinology approach. Since prokaryotic gene prediction has been shown to be highly variable (Bakke *et al.* 2009), four gene prediction programs were used to analyze the *P. abortibovis* genome. Prodigal, GeneMark, Glimmer3 (RAST), and NCBI (GeneMarkS+) predicted 1767, 2174, 2250, and 1809 genes, respectively (Table 4). A reverse vaccinology pipeline (discussed below) was used to analyze the gene sets detected by each of the gene prediction programs and the resulting vaccine candidates were subsequently compared. Ultimately, there was no significant difference in the suite of top vaccine candidates predicted using these separate gene sets. Further analysis was performed with the GeneMark gene set as it represented coverage of the largest percentage of the genome.

**Table 4:** Summary statistics of gene prediction programs

Program*^a^*	# ORFs*^b^*	Total bp	% of Genome	Average Length (bp)	% Expressed (FPKM*^c^* > 0)
Prodigal	1,767	1,512,795	83	856	56
GeneMark	2,174	1,565,122	86	720	52
Glimmer*^d^*	2,250	1,544,913	85	687	44
NCBI*^e^*	1,760	1,518,292	83	839	64

ORFs, open reading frames; FPKM, fragments per kilobase of transcript per million mapped reads; NCBI, National Center for Biotechnology Information.

a Four separate gene prediction programs were used to predict genes from the same genomic sequence.

b ORFs are sequences of nucleotides that could potentially code for proteins.

c FPKM is an RNA sequencing parameter.

d An optimized Glimmer3 was used in the RAST annotation pipeline; these are the results from the RAST output.

e NCBI gene prediction was used for the NCBI Prokaryotic Genome Annotation Pipeline; these are generated through GeneMarkS+.

**Figure 3:**
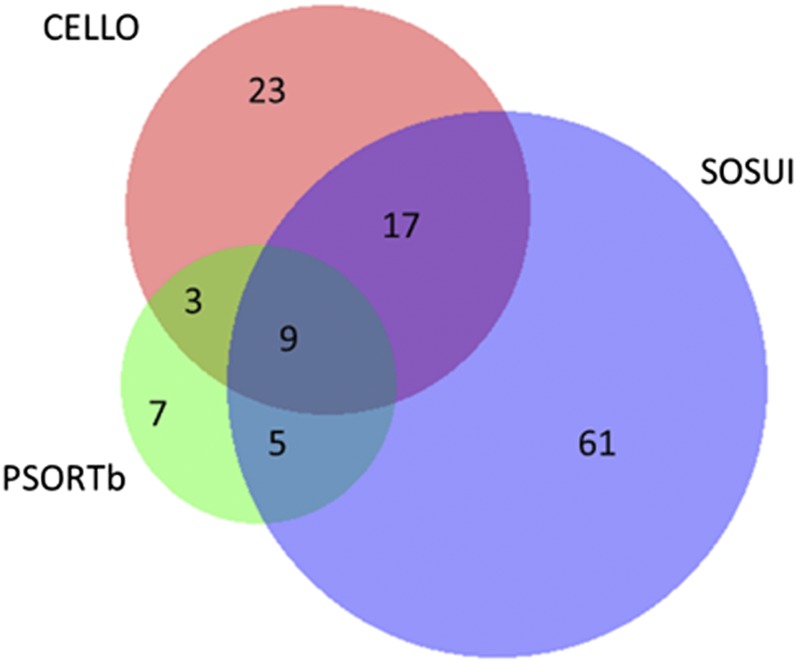
Comparison of genes that were predicted to be extracellular or outer membrane by the subcellular localization programs.

The first step in determining which ORFs represented proteins that might be antigenic was to evaluate their SCL. Extracellular or outer membrane ORFs are predicted to be more antigenic due to the accessibility of antibodies binding to these proteins. Initial comparisons were made using the programs PSORTb2.0 and PSORTb3.0; the results indicated a strong positive relationship between the programs with a Pearson’s correlation = 0.98. Therefore, only PSORTb3.0 was used in further comparisons with other (SOSUI and CELLO) SCL prediction programs. The SCL evaluation for the candidates predicted by GeneMark is summarized in Table 5, and the predicted outer membrane and extracellular proteins are summarized by a Venn diagram in Figure 3. Variation was observed between the three programs with a Pearson’s correlation of 0.28 between PSORTb and SOSUI, 0.37 between PSORTb and CELLO, and 0.37 between SOSUI and CELLO. The SCL prediction of PSORTb was the most stringent, predicting a total of only 24 extracellular or secreted proteins (Figure 3). A score of 1 was assigned for prediction of outer membrane or extracellular, 0.5 for periplasm, and 0 for cytoplasm and inner membrane. The scores of the three stand-alone SCL prediction programs (PSORTb3.0, SOSUI, and CELLO) were summed to obtain a final SCL score between 0 and 3. ORFs with an SCL score of <2 were filtered out from further analysis. This selection criterion eliminated the most ORFs, reducing the total number of ORFS from 2174 to 36 extracellular or outer membrane ORFs.

**Table 5:** Top 10 reverse vaccinology candidate list from GeneMark-predicted genes

Protein*^a^*	AA Length	Weight (kDa)	SCL*^b^*	Adhesin*^c^*	Protective*^d^*	FPKM*^e^*
00790	213	20	Extracellular	0.56	0.79	67
01995	253	27	Extracellular	0.66	0.75	10,356
03205	633	62	Extracellular	0.58	1.16	307
03255	489	49	Extracellular	0.55	1.44	138
06085	466	52	Outer Membrane	0.50	0.54	97
06335	439	47	Extracellular	0.51	0.68	156
06555	317	35	Extracellular	0.54	0.70	156
08690	550	59	Extracellular	0.54	0.70	60
08695	561	60	Extracellular	0.63	0.76	880
08860	108	8	Extracellular	0.65	0.66	—

AA, amino acid; SCL, subcellular localization; FPKM, fragments per kilobase of transcript per million mapped reads; NCBI, National Center for Biotechnology Information.

a Protein numbering is based on the NCBI Prokaryotic Genome Annotation Pipeline from the DNA accession number (CP016908) in GenBank; the nomenclature for all proteins is preceded by BCY86_.

b SCL is a consensus from the three standalone programs used PSORTb3.0, SOSUI, and CELLO.

c Adhesin is the probability of a protein being an adhesin protein and is predicted by SPAAN from the Vaxign pipeline.

d Protective is a score given by the program VaxiJen, which predicts the likelihood that a protein is a protective antigen.

e FPKM is a parameter to quantify RNA sequencing data.

The number of TM helices was calculated for each of the extracellular or outer membrane ORFs, and one candidate of the 36 was removed from the analysis because it had two or more TM domains. Next, ORFs that shared homology with eukaryotic proteins (BLAST *E* < 10^−5^) were removed from the analysis. Of the 2174 total ORFs, 406 (19%) were highly conserved between *P. abortibovis* and eukaryotes, including two of the remaining 35 extracellular or outer membrane candidates. Four candidates that shared further protein sequence homology with *S. cellulosum* were also removed from subsequent analysis. Finally, the adhesin probability was computed for all ORFs with only 59 out of 2174 (2.7%) ORFs predicted to be adhesins (*P*_ad_ > 0.5). Only 2.7% (59 of 2174) of the total genes met the threshold and hence this step reduced the *P. abortibovis* candidates by almost two-thirds, from 29 candidates to 10.

The protective antigenicity of each candidate was computed with the VaxiJen score and was filtered on a predictive score of 0.5. The default score of 0.4 was too liberal, with 1487 of 2174 (68%) ORFs predicted to be protective antigens, whereas a cutoff of 0.5 predicted 890 of 2174 (41%) candidates.

The annotated biological functions of these proteins were evaluated to determine if they had sequence homology to proteins known to be antigenic. The top 10 candidates were predicted to be hypothetical proteins with no significant homology to proteins of known function. The last parameter that was used in the selection of vaccine candidates was the FPKM value obtained from RNA-Seq analysis. Nine of the 10 candidates were expressed in our RNA-Seq results (Table 4) and these FPKM values were used to rank among these top nine candidates.

### Immunological testing

Nine of 10 vaccine candidate proteins were successfully expressed as 6 × His fusion proteins in *E. coli*. Protein *BCY86_08860* was not successfully expressed and was removed from further analysis. Mouse anti-6 × His bound eight of nine expressed proteins (Table 6). One protein, *BCY86_03205*, was not detected by immunoblotting when probed with 6 × His antibody and was removed from further analysis. One protein (*BCY86_06555*) reacted only to serum from control mice, and will not be discussed further. The remaining seven candidate proteins were bound by serum from EBA-infected mice. Three protein candidates, *BCY86_00790*, *BCY86_01995*, and *BCY86_08695*, were specifically bound by serum derived only from EBA-infected mice. Three additional proteins, *BCY86_06335*, *BCY86_03255*, and *BCY86_08690*, were more strongly bound by the serum from EBA-infected mice than the serum from the control mice. Protein *BCY86_06085* was equally bound by serum derived from both the positive EBA-infected mice and the control mice, and appeared to have an additional protein band bound at ∼100 kDa of unknown origin.

**Table 6:** Immunological testing through western blot analysis on the top nine protein candidates

			Murine		Bovine Cow		Bovine Fetus	
Protein*^a^*	MWT (kDa)*^b^*	Anti-His	EBA+	Neg	EBA+	Neg	EBA+	Neg
00790	25	+	+	−	+	±	+	−
01995	37	+	+	−	+	−	+	−
03205	65	−	NA	NA	NA	NA	NA	NA
03255	55	+	+	±	+	±	±	−
06085	45	+	+	+	+	±	+	−
06335	47	+	+	±	±	−	−	−
06555	37	+	−	+	−	±	−	−
08690	60	+	+	±	±	±	±	−
08695	58	+	+	−	−	−	−	−

MWT, molecular weight; His, histidine; EBA, epizootic bovine abortion; Neg, negative; +, clear binding of antibodies to the protein candidate; −, no presence of antibody binding; ±, weak binding of antibody to the protein candidate. NA, not analyzed.

a Protein numbering is based on the NCBI prokaryotic annotation pipeline from the DNA accession number (CP016908) in GenBank; the nomenclature for all proteins is preceded by BCY86_.

b The observed molecular weight from western blots.

Results using serum from mature cows were similar to those of the mouse with the exception of *BCY86_08695*, which showed no binding to serum from EBA-infected or control cows. Protein *BCY86_01995* displayed specific binding to serum from only the EBA-infected cow and not the control. Proteins *BCY86_00790*, BCY86_03255, *BCY86_06085*, and *BCY86_06335* were more strongly bound by the serum from the EBA-infected cow than the serum from the control cow. Protein BCY86_08690 showed equivalent binding to serum from both the EBA-infected and the negative cow, suggesting it was not specific.

The serum from EBA-infected fetuses specifically bound proteins *BCY86_01995*, *BCY86_06085*, and *BCY86_00790* while serum from control fetuses did not, whereas *BCY86_03255* and *BCY86_08690* showed potential antigenic responses indicated by a weak response in serum from the EBA-positive fetus and no binding to serum from the negative fetus.

In summary, protein *BCY86_01995* was the most promising candidate, displaying a strong specific immune response in EBA-infected mouse, cow, and bovine fetal serum. Protein *BCY86_00790* specifically reacted with EBA-infected mouse and bovine fetal serums only and reacted more strongly with infected cow serum than the control cow serum suggesting it is also a good vaccine candidate. Protein *BCY86_03255* also displayed stronger antibody binding with the EBA-infected serums compared to control serums of both murine and bovine origin.

## Discussion

The *de novo* assembly of the *P. abortibovis* genome was complicated by the presence of eukaryotic DNA from the spleen cells of the SCID mice in which the bacteria were grown. Although the single-cell suspension was enriched for bacterial cells, the bacterium resides intracellularly and cannot be purified from the mouse DNA. We hypothesized that deep coverage NGS and bioinformatics analysis would overcome the eukaryotic DNA contamination and enable the assembly of the complete bacterial genome.

The completion of the circularized Deltaproteobacterial genome confirmed this hypothesis. The resulting sequence showed that *P. abortibovis* belongs to the order Myxococcales, which includes species with the largest known bacterial genomes. The closest phylogenetic relative to *P. abortibovis* that has been sequenced is *S. cellulosum*, a myxobacterium that resides in the soil and has the largest known bacterial genome at 13 Mb. Although these organisms have highly divergent lifestyles, comparative functional analysis revealed high levels of conservation among functional roles as well as protein, AA, and DNA sequences between the two species. Although the assembled *P. abortibovis* genome is <<13 Mb, research indicates that when bacterial lineages transition from free-living organisms to permanently living in a host (*i.e.*, obligate pathogens), they rely on the host environment for many essential proteins and thereafter substantially reduce their genome size (Moran and Plague 2004).

Moran (2002) established that the compounds of intermediate metabolism and AAs can be obtained from host cells and thus are a clear basis for genome reduction in host-associated bacteria. This can clearly be seen in *P. abortibovis*. When the functional groups (Figure 2) contributing to intermediate metabolism (potassium metabolism, iron metabolism, secondary metabolism, nitrogen metabolism, aromatic metabolism, sulfur metabolism, and phosphorus metabolism) are summed, *S. cellulosum* has 172 functional roles whereas *P. abortibovis* has only 12, a 93% reduction in the genes that code for intermediate metabolism. This reduction far exceeds that observed in any other functional group. The genes that benefit free-living organisms and their need to survive in extreme environmental conditions, such as regulatory elements, are also lost. This phenomenon is supported in *P. abortibovis* with a 94% reduction in the genes responsible for regulatory elements and cell signals. DNA repair enzymes are reduced in *P. abortibovis* by 54%, from 58 to 27. The loss of DNA repair enzymes has a substantial impact on the genome size reduction and the adenine (A)–thymine (T) bias observed in most host-associated genomes.

In bacteria, genome size and G–C content are correlated (*r* = 0.46) (Nishida 2012) and the obligate host-associated bacteria contain genomes with relatively lower G–C content. The genomes with the highest observed A–T enrichment are bacterial symbionts with extremely small genomes. For example, the Candidatus *Zinderia insecticola* has a 209 kbp genome with a G–C content of only 13.5% (McCutcheon and Moran 2012). This relationship is also observed when comparing *P. abortibovis* (47.2% G–C content) and *S. cellulosum* (72% G–C content). The existence of an inherent mutational bias from G–C to A–T was observed experimentally and can likely be attributed to DNA damage, such as cytosine deamination and guanine oxidation, which results in G–C being converted to A–T. This further suggests the inability of *P. abortibovis* to survive outside a host environment. The phenomenon of genome reduction and A–T bias is further supported with the most closely related pathogen to *P. abortibovis*, *L. intracellularis*, which contains 1.72 Mbp of genomic DNA. This Deltaproteobacterium contains a 1.46 Mbp circular genome (33% G–C content) and three plasmids that are 27.1, 39.9, and 194 kbp (29, 29, and 33% G–C content, respectively) in size (Sait *et al.* 2013).

Although reduced genomes do not have a universal set of genes that are maintained, there are patterns associated with the types of genes that are lost (Moran 2002). Of the many genomic sequences that have been assembled, only obligate intracellular pathogens and symbionts have a stronger evolutionary force with genome reduction than horizontal gene transfer (Lawrence 2005). Therefore, it is reasonable to hypothesize that *P. abortibovis* has evolved to become an obligate eukaryotic host-associated organism that does not contain an environmental reservoir outside eukaryotic organisms.

Understanding the evolution of microbes and the pathogenesis of disease can guide vaccine candidate selection. Reverse vaccinology is the utilization of genomic sequence to make *in silico* predictions of vaccine candidates. This process has evolved to further refine the selection criteria for a smaller group of candidates to be experimentally tested in immunologic assays. The *in silico* processing is dependent on the genes predicted and their resulting AA sequences. High variation is generally observed with the prediction of ORFs from a raw nucleotide sequence.

In this study, although the overall variation between predicted genes was high across each gene prediction program (Table 3), the relative variation was minimized after the prediction of vaccine candidates (Table 4). Slight differences observed in the prediction of vaccine candidates with each set of genes did not ultimately change the candidates identified by reverse vaccinology.

The main source of candidate filtering in reverse vaccinology was identification of outer membrane and secreted proteins from the prediction of SCL. Selection criteria was stringent to ensure that the properties encoded by the AA sequence represented a true secreted or outer membrane protein. The *in silico* identification of outer membrane proteins was successfully utilized for antigen discovery in the intracellular Deltaproteobacterial pathogen *L. intracellularis* (Watson *et al.* 2011). Although these proteins were discovered through proteomic analysis, the results of this study support the utilization of subcellular localization to identify antigenic proteins in *P. abortibovis*.

The next criterion for selection was one TM domain or less for ease of protein expression. The first reverse vaccinology experiment was performed on *Neisseria meningitidis* serogroup B (MenB), which only utilized *in silico* analysis for SCL. This experiment attempted to express the proteins encoded by 570 genes and found that the number of TM domains was inversely correlated with the ability to express the protein (Pizza *et al.* 2000). This parameter has been adopted for further RV projects and resulted in the removal of one candidate ORF in this study.

The sequence similarity of predicted candidates to proteins in other organisms is an important parameter to consider for vaccine development. The candidate should not have sequence similarity with the host to prevent autoimmunity and to ensure that the host recognizes the protein as foreign so that it can mount an immune response (He *et al.* 2010). Protective antigens generally do not share homology with proteins of any eukaryotic or highly conserved genes.

Reverse vaccinology has proven beneficial for organisms with a high degree of strain divergence because homology analysis can filter proteins likely to display strain-specific immunity (Pizza *et al.* 2000). Therefore, the ideal candidate would be conserved in all pathogenic strains and be nonhomologous to any proteins in commensal strains. However, strain-specific proteins can still be included in cocktail vaccines if they trigger a significant immune response in that strain. This has been seen with the inclusion of two proteins (GNA1030 and GNA2091) in the Bexsero vaccine for meningococcal B (Giuliani *et al.* 2006).

Protective immunogenicity is the primary goal for any subunit vaccine. The host immune system can mount an adaptive immune response that recognizes a vast spectrum of microbial immunogens with no apparent host-protective antigenicity. Therefore, vaccine design targets those antigens that can induce immune responses that are protective. In this study, the program VaxiJen was used to supplement the other RV programs to better predict whether the candidates will result in a protective immune response.

The FPKM obtained from the Cufflinks analysis was utilized as the main ranking criterion for protein expression. The presence of RNA expression from the candidates also confirms that these predicted proteins are not artifacts of the gene prediction algorithms. Visualization of the RNA-Seq reads also allows for more precise gene boundary assignment and possible reassignment of start and stop sites.

Of the 10 final candidates, one of the proteins was located on the outer membrane and the rest were predicted to be secreted. The probability of a protein being an adhesin was relatively consistent between the candidates (∼0.6) but the protective antigenicity score was highly variable (0.54–1.44), allowing for ranking of candidates based on their protective antigenicity score. Finally, candidates were evaluated by their FPKM value, which designates the level of *in vivo* murine transcription for that gene. The RNA-Seq experiment was conducted in SCID mice and might not represent the expression levels in the target host (pregnant cow) during infection. The top candidate represents a gene that is highly expressed, being among the top 15 expressed ORFs. This transcript has even coverage of RNA-Seq reads spanning the length of the transcript, indicating that the gene coordinates were properly assigned.

The screening of vaccine candidates against serums of EBA-infected mice and negative control sera provides an initial understanding of the relative antigenicity of the protein candidates. Evaluation of antigen binding to antibodies derived from EBA-infected mice provided a broad picture of the potential for these proteins to induce an immune response, with seven of nine expressed proteins displaying a positive reaction (Table 5). The mouse provides the widest range antibody profile, possibly due to the fact that the antibodies are representative of the immune responses from a pool of four mice. The two top candidates from reverse vaccinology, *BCY86_01995* and *BCY86_08695*, were bound by murine-derived antibodies. Four additional proteins showed binding to murine-derived antibodies, but some binding to antibodies from naïve control mice was evident. One protein displayed equal binding with the positive and negative serums. This indicates that there is a cross-reactive response of the protein with potential epitopes to other microorganisms.

The antibody-binding profile of the bovine serum better reflects the true potential of these candidate proteins for a recombinant vaccine. The antibody-binding profile was similar between the mouse and the bovine sera with the exception of *BCY86_08695*, which showed no binding to bovine immune serum antibodies. Protein *BCY86_01995* demonstrated specific binding to antibody from bovine immune serum. The remaining proteins were bound by antibodies from both immune and naïve cattle, though not necessarily to the same degree. Four proteins were more strongly bound by antibodies from immune bovine serum as compared to the naïve counterpart. These apparent nonspecific reactions can likely be attributed to cross-reactivity of the recombinant proteins with other bacteria. Cows are exposed to many pathogenic and nonpathogenic microorganisms, and therefore have a broad antibody profile. Serum antibodies from aborted fetuses bound three proteins (*BCY86_00790*, *BCY86_01995*, and *BCY86_06085*) with specific antigenic responses and displayed minimal binding with two additional proteins (*BCY86_03255* and *BCY86_08690*). The negative control fetal serum did not show binding with any recombinant proteins. This is likely due to the absence of microbe-specific antibodies in a healthy fetus.

The functional immunological testing of these protein candidates provides an initial validation of the reverse vaccinology *in silico* vaccine candidate prediction approach. The demonstration of an antibody response only addresses the humoral arm of the immune system and evidence of immunogenicity but does not validate actual protection. Robust antibody responses to intracellular pathogens often do not equate to protection. Further immunogenicity studies (ability to induce a specific antibody response in the host) need to be performed to determine the potential of these proteins to be viable as a recombinant vaccine. The results of this study indicate that the seven proteins displaying antigenic responses are promising targets for further studies in the development of a protective subunit recombinant vaccine for EBA.

In summary, protein *BCY86_01995* displayed specific binding of antibodies derived from all three immune serum types (mature bovine, fetal bovine, and murine), providing evidence for designation as an EBA-specific antigen in the target bovine species. *In silico* analysis of this protein through the reverse vaccinology pipeline clearly ranked this protein as one of the top two vaccine candidates. Protein *BCY86_01995* was ranked as the most promising candidate based on RNA-Seq results, which revealed an expression level that was an order of magnitude higher than the next candidate in the list. The other protein ranked in the top two, *BCY86_08695*, showed a positive response with EBA-specific binding in the mouse but did not exhibit any binding in the cow serum samples. The functional immunological testing of the top two candidates from reverse vaccinology with antigenicity specifically in serum from EBA-infected mice supports this approach as a useful tool for immunologists to narrow their pool of potential vaccine candidates.

## Supplementary Material

Supplemental material is available online at www.g3journal.org/lookup/suppl/doi:10.1534/g3.116.036673/-/DC1.

## References

[bib1] Altschul S. F. Gish W. Miller W. Myers E. W. Lipman D. J., 1990 Basic local alignment search tool. J. Mol. Biol. 215: 403–410. 2231712 10.1016/S0022-2836(05)80360-2

[bib2] Anderson M. L. Kennedy P. C. Blanchard M. T. Barbano L. Chiu P., 2006 Histochemical and immunohistochemical evidence of a bacterium associated with lesions of epizootic bovine abortion. J. Vet. Diagn. Invest. 18: 76–80. 16566260 10.1177/104063870601800110

[bib3] Aziz R. K. Bartels D. Best A. A. DeJongh M. Disz T., 2008 The RAST Server: rapid annotations using subsystems technology. BMC Genomics 9: 75. 18261238 10.1186/1471-2164-9-75 PMC2265698

[bib4] Bakke P. Carney N. DeLoache W. Gearing M. Ingvorsen K., 2009 Evaluation of three automated genome annotations for *Halorhabdus utahensis*. PLoS One 4: e6291. 19617911 10.1371/journal.pone.0006291 PMC2707008

[bib5] Besemer J. Lomsadze A. Borodovsky M., 2001 GeneMarkS: a self-training method for prediction of gene starts in microbial genomes. Implications for finding sequence motifs in regulatory regions. Nucleic Acids Res. 29: 2607–2618. 11410670 10.1093/nar/29.12.2607 PMC55746

[bib6] Bigelow H. R. Petrey D. S. Liu J. Przybylski D. Rost B., 2004 Predicting transmembrane beta-barrels in proteomes. Nucleic Acids Res. 32: 2566–2577. 15141026 10.1093/nar/gkh580 PMC419468

[bib7] Blanchard M. T. Chen C. I. Anderson M. Hall M. R. Barthold S. W., 2010 Serial passage of the etiologic agent of epizootic bovine abortion in immunodeficient mice. Vet. Microbiol. 144: 177–182. 20144513 10.1016/j.vetmic.2010.01.002

[bib8] Blanchard M. T. Anderson M. L. Hoar B. R. Pires A. F. Blanchard P. C., 2014 Assessment of a fluorescent antibody test for the detection of antibodies against epizootic bovine abortion. J. Vet. Diagn. Invest. 26: 622–630. 25139792 10.1177/1040638714545506

[bib9] Boetzer M. Henkel C. V. Jansen H. J. Butler D. Pirovano W., 2011 Scaf-folding pre-assembled contigs using SSPACE. Bioinformatics 27: 578–579. 21149342 10.1093/bioinformatics/btq683

[bib10] Brooks R. S. Blanchard M. T. Anderson M. L. Hall M. R. Stott J. L., 2011 Quantitative duplex TaqMan real-time polymerase chain reaction for the assessment of the etiologic agent of epizootic bovine abortion. J. Vet. Diagn. Invest. 23: 1153–1159. 22362796 10.1177/1040638711425573

[bib11] Brooks R. S. Blanchard M. T. Clothier K. A. Fish S. Anderson M. L., 2016 Characterization of *Pajaroellobacter abortibovis*, the etiologic agent of epizootic bovine abortion. Vet. Microbiol. 192: 73–80. 27527767 10.1016/j.vetmic.2016.07.001

[bib12] Delcher A. L. Bratke K. A. Powers E. C. Salzberg S. L., 2007 Identifying bacterial genes and endosymbiont DNA with Glimmer. Bioinformatics 23: 673–679. 17237039 10.1093/bioinformatics/btm009 PMC2387122

[bib13] Doytchinova I. A. Flower D. R., 2007 VaxiJen: a server for prediction of protective antigens, tumour antigens and subunit vaccines. BMC Bioinformatics 8: 4. 17207271 10.1186/1471-2105-8-4 PMC1780059

[bib14] Ellis R. W., 1999 New technologies for making vaccines. Vaccine 17: 1596–1604. 10194811 10.1016/s0264-410x(98)00416-2

[bib15] Giuliani M. M. Adu-Bobie J. Comanducci M. Aricò B. Savino S., 2006 A universal vaccine for serogroup B meningococcus. Proc. Natl. Acad. Sci. USA 103: 10834–10839. 16825336 10.1073/pnas.0603940103 PMC2047628

[bib16] He Y. Xiang Z. Mobley H. L., 2010 Vaxign: the first web-based vaccine design program for reverse vaccinology and applications for vaccine development. J. Biomed. Biotechnol. 2010: 297505. 20671958 10.1155/2010/297505 PMC2910479

[bib17] Howarth J. A. Moulton J. E. Frazier L. M., 1956 Epizootic bovine abortion characterized by fetal hepatopathy. J. Am. Vet. Med. Assoc. 128: 441–449. 13306666

[bib18] Hyatt D. Chen G. L. Locascio P. F. Land M. L. Larimer F. W., 2010 Prodigal: prokaryotic gene recognition and translation initiation site identification. BMC Bioinformatics 11: 119. 20211023 10.1186/1471-2105-11-119 PMC2848648

[bib19] Imai K. Asakawa N. Tsuji T. Akazawa F. Ino A., 2008 SOSUI-GramN: high performance prediction for sub-cellular localization of proteins in gram-negative bacteria. Bioinformation 2: 417–421. 18795116 10.6026/97320630002417 PMC2533062

[bib20] Kearse M. Moir R. Wilson A. Stones-Havas S. Cheung M., 2012 Geneious Basic: an integrated and extendable desktop software platform for the organization and analysis of sequence data. Bioinformatics 28: 1647–1649. 22543367 10.1093/bioinformatics/bts199 PMC3371832

[bib21] Kennedy P. C. Casaro A. P. Kimsey P. B. Bon Durant R. H. Bushnell R. B., 1983 Epizootic bovine abortion: histogenesis of the fetal lesions. Am. J. Vet. Res. 44: 1040–1048. 6870006

[bib22] King D. P. Chen C. I. Blanchard M. T. Aldridge B. M. Anderson M., 2005 Molecular identification of a novel deltaproteobacterium as the etiologic agent of epizootic bovine abortion (foothill abortion). J. Clin. Microbiol. 43: 604–609. 15695652 10.1128/JCM.43.2.604-609.2005 PMC548036

[bib23] Langmead B. Salzberg S. L., 2012 Fast gapped-read alignment with Bowtie 2. Nat. Methods 9: 357–359. 22388286 10.1038/nmeth.1923 PMC3322381

[bib24] Lawrence J. G., 2005 Common themes in the genome strategies of pathogens. Curr. Opin. Genet. Dev. 15: 584–588. 16188434 10.1016/j.gde.2005.09.007

[bib25] Li L. Stoeckert C. J. Jr. Roos D. S., 2003 OrthoMCL: identification of ortholog groups for eukaryotic genomes. Genome Res. 13: 2178–2189. 12952885 10.1101/gr.1224503 PMC403725

[bib26] McCutcheon J. P. Moran N. A., 2012 Extreme genome reduction in symbiotic bacteria. Nat. Rev. Microbiol. 10: 13–26. 10.1038/nrmicro2670 22064560

[bib27] Moran N. A., 2002 Microbial minimalism: genome reduction in bacterial pathogens. Cell 108: 583–586. 11893328 10.1016/s0092-8674(02)00665-7

[bib28] Moran N. A. Plague G. R., 2004 Genomic changes following host restriction in bacteria. Curr. Opin. Genet. Dev. 14: 627–633. 15531157 10.1016/j.gde.2004.09.003

[bib29] Mouse Genome Sequencing Consortium Waterston R. H. Lindblad-Toh K. Birney E. Rogers J., 2002 Initial sequencing and comparative analysis of the mouse genome. Nature 420: 520–562. 12466850 10.1038/nature01262

[bib30] Nadalin F. Vezzi F. Policriti A., 2012 GapFiller: a de novo assembly approach to fill the gap within paired reads. BMC Bioinformatics 13(Suppl. 14): S8. 10.1186/1471-2105-13-S14-S8 PMC3439727 23095524

[bib31] Nishida H., 2012 Evolution of genome base composition and genome size in bacteria. Front. Microbiol. 3: 420. 23230432 10.3389/fmicb.2012.00420 PMC3515811

[bib32] Overbeek R. Olson R. Pusch G. D. Olsen G. J. Davis J. J., 2014 The SEED and the rapid annotation of microbial genomes using subsystems technology (RAST). Nucleic Acids Res. 42: D206–D214. 24293654 10.1093/nar/gkt1226 PMC3965101

[bib33] Pizza M. Scarlato V. Masignani V. Giuliani M. M. Aricò B., 2000 Identification of vaccine candidates against serogroup B meningococcus by whole-genome sequencing. Science 287: 1816–1820. 10710308 10.1126/science.287.5459.1816

[bib34] Rappuoli R., 2001 Reverse vaccinology, a genome-based approach to vaccine development. Vaccine 19: 2688–2691. 11257410 10.1016/s0264-410x(00)00554-5

[bib35] Sachdeva G. Kumar K. Jain P. Ramachandran S., 2005 SPAAN: a software program for prediction of adhesins and adhesin-like proteins using neural networks. Bioinformatics 21: 483–491. 15374866 10.1093/bioinformatics/bti028 PMC7109999

[bib36] Sait M. Aitchison K. Wheelhouse N. Wilson K. Lainson F. A., 2013 Genome sequence of *Lawsonia intracellularis* strain N343, isolated from a sow with hemorrhagic proliferative enteropathy. Genome Announc. 1: e00027-13. 10.1128/genomeA.00027-13 PMC3587925 23472224

[bib37] Sambrook, J., and D. W. Russell, 2006 Purification of nucleic acids by extraction with phenol:chloroform. CSH Protoc. 2006. Available at: http://cshprotocols.cshlp.org/content/2006/1/pdb.prot4455.long. 10.1101/pdb.prot4455 22485786

[bib38] Schmidtmann E. T. Bushnell R. B. Loomis E. C. Oliver M. N. Theis J. H., 1976 Experimental and epizootiologic evidence associating *Ornithodoros coriaceus* Koch (Acari-Argasidae) with exposure of cattle to epizootic bovine abortion in California. J. Med. Entomol. 13: 292–299. 1011232 10.1093/jmedent/13.3.292

[bib39] Tatusova T. A. Madden T. L., 1999 BLAST 2 Sequences, a new tool for comparing protein and nucleotide sequences. FEMS Microbiol. Lett. 174: 247–250. 10339815 10.1111/j.1574-6968.1999.tb13575.x

[bib40] Tatusova, T., M. DiCuccio, A. Badretdin, V. Chetvernin, S. Ciufo *et al.* 2013 Prokaryotic genome annotation pipeline. in *The NCBI Handbook*, Ed. 2. National Center for Biotechnology Information, Bethesda, MD. Available at: https://www.ncbi.nlm.nih.gov/books/NBK174280/.

[bib41] Tatusova T. DiCuccio M. Badretdin A. Chetvernin V. Nawrocki E. P., 2016 NCBI prokaryotic genome annotation pipeline. Nucleic Acids Res. 44: 6614–6624. 27342282 10.1093/nar/gkw569 PMC5001611

[bib42] Teglas M. B. Drazenovich N. L. Stott J. Foley J. E., 2006 The geographic distribution of the putative agent of epizootic bovine abortion in the tick vector, *Ornithodoros coriaceus*. Vet. Parasitol. 140: 327–333. 16672178 10.1016/j.vetpar.2006.03.027

[bib43] Trapnell C. Hendrickson D. G. Sauvageau M. Goff L. Rinn J. L., 2013 Differential analysis of gene regulation at transcript resolution with RNA-seq. Nat. Biotechnol. 31: 46–53. 23222703 10.1038/nbt.2450 PMC3869392

[bib44] Untergasser A. Cutcutache I. Koressaar T. Ye J. Faircloth B. C., 2012 Primer3–new capabilities and interfaces. Nucleic Acids Res. 40: e115. 22730293 10.1093/nar/gks596 PMC3424584

[bib45] Watson E. Clark E. M. Alberdi M. P. Inglis N. F. Porter M., 2011 A novel *Lawsonia intracellularis* autotransporter protein is a prominent antigen. Clin. Vaccine Immunol. 18: 1282–1287. 21697340 10.1128/CVI.05073-11 PMC3147349

[bib46] Yu C. S. Chen Y. C. Lu C. H. Hwang J. K., 2006 Prediction of protein subcellular localization. Proteins 64: 643–651. 16752418 10.1002/prot.21018

[bib47] Yu N. Y. Wagner J. R. Laird M. R. Melli G. Rey S., 2010 PSORTb 3.0: improved protein subcellular localization prediction with refined localization subcategories and predictive capabilities for all prokaryotes. Bioinformatics 26: 1608–1615. 20472543 10.1093/bioinformatics/btq249 PMC2887053

[bib48] Zerbino D. R. Birney E., 2008 Velvet: algorithms for de novo short read assembly using de Bruijn graphs. Genome Res. 18: 821–829. 18349386 10.1101/gr.074492.107 PMC2336801

