# Supplementary figures and images for: Genome Report: Identification and Validation of Antigenic Proteins from Pajaroellobacter abortibovis Using De Novo Genome Sequence Assembly and Reverse Vaccinology
Source: G3 (Bethesda). 2016 Dec 28;7(2):321–31. doi: 10.1534/g3.116.036673 (PMC5295582; doi:10.1534/g3.116.036673)

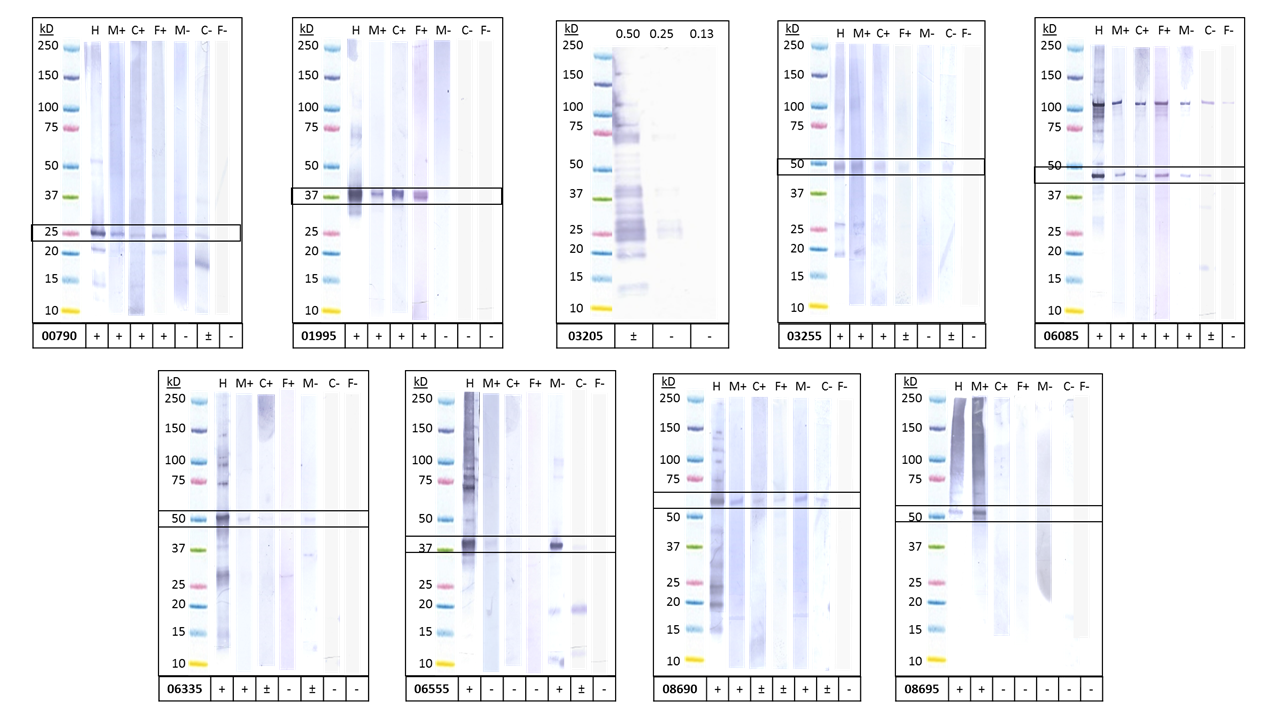

Supplement: Supplementary file 1 [file 321FigureS1.tif]
