# Supplementary material for: Genome Report: Identification and Validation of Antigenic Proteins from Pajaroellobacter abortibovis Using De Novo Genome Sequence Assembly and Reverse Vaccinology
Source: G3 (Bethesda). 2016 Dec 28;7(2):321–31. doi: 10.1534/g3.116.036673 (PMC5295582; doi:10.1534/g3.116.036673)
Supplement: Supplementary file 2 [file 321TableS1.docx]

Table S1. RNA-Seq expression from NCBI prokaryotic annotation pipeline predicted genes. (.xlsx, 48.4 KB)

Available for download at

<http://www.g3journal.org/lookup/suppl/doi:10.1534/g3.116.036673/-/DC1/TableS1.xlsx>
